# Fire and ant interactions mediated by honeydew and extrafloral nectar in an australian tropical savanna

**DOI:** 10.1007/s00442-024-05628-6

**Published:** 2024-10-05

**Authors:** Fernanda M. P. Oliveira, Carlos H. F. Silva, Melinda L. Moir, Inara R. Leal, Alan N. Andersen

**Affiliations:** 1 Programa de Pós-Graduação em Biologia Vegetal, Universidade Federal de Pernambuco, Recife, Brazil; 2 Department of Primary Industries and Regional Development, South Perth, WA Australia; 3 Departamento de Botânica, Universidade Federal de Pernambuco, Recife, Brazil; 4 Research School for the Environment and Livelihoods, Charles Darwin University, Darwin, NT Australia

**Keywords:** Disturbance, Dominant ants, Experimental burning, Hemipteran interactions, Mutualism

## Abstract

Fire is a major disturbance affecting ecosystems globally, but its impact on mutualisms has received minimal attention. Here, we use a long-term field experiment to investigate the impact of different fire regimes on globally important ant–honeydew and ant–extrafloral nectar (EFN) mutualistic interactions in an Australian tropical savanna. These interactions provide ants with a key energy source, while their plant and hemipteran hosts receive protection services. We examined ant interactions on species of *Eucalyptus* (lacking EFNs) and *Acacia* (with EFNs) in three replicate plots each of burning every 2 and 3 years early in the dry season, burning late in the dry season every 2 years, and unburnt for > 25 years. The proportions of plants with ant–honeydew interactions in *Acacia* (44.6%) and *Eucalyptus* (36.3%) were double those of *Acacia* plants with ant–EFN interactions (18.9%). The most common ants, representing 85% of all interactions, were behaviourally dominant species of *Oecophylla*, *Iridomyrmex* and *Papyrius*. Fire promoted the incidence of ant interactions, especially those involving EFNs on *Acacia*, which occurred on only 3% of plants in unburnt plots compared with 24% in frequently burnt plots. Fire also promoted the relative incidence of behaviourally dominant ants, which are considered the highest quality mutualists. Contrary to expectations, frequent fire did not result in a switching of behaviourally dominant ant partners from forest-adapted *Oecophylla* to arid-adapted *Iridomyrmex*. Our findings that frequent fire increases ant interactions mediated by honeydew and extrafloral nectar, and promotes the quality of ant mutualists, have important implications for protective services provided by ants in highly fire-prone ecosystems.

**Supplementary Information:**

The online version contains supplementary material available at 10.1007/s00442-024-05628-6.

## Introduction

Mutualisms—interspecific cooperative interactions in which both partners benefit (Bronstein 2009; Janzen 1975), are essential for the origin and ongoing maintenance of biodiversity (Andresen et al. 2018; Bronstein et al. 2006; Thompson 1999; Tylianakis et al. 2008). A range of essential ecosystem services such as pollination, seed dispersal and cycling of nutrients are the result of mutualistic interactions (Potts et al. 2010; Terborgh et al. 2008, Wilson et al. 2009). However, we have a limited understanding of how mutualist interactions respond to disturbances, either natural or anthropogenic (Teixido et al. 2022; Vasconcelos et al. 2020; Vidal et al. 2021).

Ants are an ecologically dominant faunal group globally that feature in many mutualistic interactions (Bronstein 2015; Rico-Gray and Oliveira 2007). Such interactions include the provision of protection services to partners that provide food resources in the form of liquid carbohydrate (Davidson 1997; Hölldobler and Wilson 1990). Chief among these carbohydrate resources is honeydew produced by hemipteran insects (Blüthgen et al. 2000; Del-Claro 2004) and plant secretions from extrafloral nectaries (EFNs) (Blüthgen et al. 2000; Davidson et al. 2003). Both honeydew and extrafloral nectar specifically attract ants (Bentley 1977; Blüthgen et al. 2000), which then repel or kill insect predators and herbivores (Del-Claro et al. 2016; Heil 2015; Zhang et al. 2012), potentially increasing the growth and reproductive success of the hemipteran and plant partners (Nascimento and Del-Claro 2010; Styrsky and Eubanks 2010). Conversely, liquid carbohydrate is a key resource for ants, especially for behaviourally dominant species that require it for fuelling their large colony sizes, high rates of activity and aggressive behaviour (Blüthgen et al. 2000; Davidson et al. 2004; Holway et al. 2002; Lach et al. 2020). Honeydew is an especially valuable resource because it can be produced continuously in large quantities that are predictable in space and time (Blüthgen et al. 2000, Fiala, 1990, Rico-Gray and Oliveira 2007). Moreover, it contains a broader spectrum of sugars than does extrafloral nectar (Blüthgen et al. 2004a). Honeydew tends to attract behaviourally dominant dolichoderines and formicines that possess adaptations for processing large quantities of liquid carbohydrate, and such behaviourally dominant species provide the best protection services because of their aggressive behaviour (Blüthgen et al. 2000; Davidson et al. 2004; Holway et al. 2002; Lach et al. 2020). In contrast, EFNs often attract behaviourally subordinate species that tend them opportunistically (Blüthgen et al. 2004b; Blüthgen and Fiedler 2004; Davidson et al. 2003).

Fire is a frequent and widespread disturbance shaping the biota and functioning of many ecosystems around the globe (Andersen et al. 2005; Bond et al. 2005; Bowman et al. 2009; Brando et al. 2019). The impact of fire on ants has been extensively studied (Vasconcelos et al. 2017; Andersen 2019), but little attention has been given to the effects of fire on the mutualistic relationships between ants and other organisms. The effects of fire on ants, and especially on the abundance of behaviourally dominant species, have important implications for the protection services they provide. For example, a negative effect of severe fire on the outcome of mutualistic interactions mediated by EFNs was attributed to changes in ant communities, particularly a reduction in the abundance of arboreal specialists (Vasconcelos et al. 2020). On the other hand, fire can also affect these mutualisms through stress-induced increases in the quality and quantity of nectar produced by EFNs, thus promoting ant attendance (Alves-Silva and Del-Claro 2013; Silva et al. 2020). Similarly, potential hemipteran partners can be sensitive to different burning regimes. For example, experimental burning in an Australian savanna increased the overall abundance of plant-dwelling ‘Homoptera’ (Sternorrhyncha + Auchenorrhyncha; Andersen and MÜller 2000).

Here, we use a long-term fire experiment in an Australian tropical savanna to examine the effects of different fire regimes on ant–honeydew and ant–EFN interactions as an indicator of mutualistic outcomes. Australian savannas are among the world’s most fire-prone biomes. Here, frequent fire promotes ant abundance and diversity because it produces an open habitat that is strongly favoured by the predominantly arid-adapted fauna (Andersen and Vasconcelos 2022). Australian savannas feature two of the world’s most behaviourally dominant ant genera in *Iridomyrmex* and *Oecophylla*, which have contrasting responses to fire (Andersen et al. 2007). *Iridomyrmex* is an arid-adapted ground-nesting genus that prefers open habitats; it suffers little direct mortality during fire because of the protection afforded by its soil nests, and its preferred open habitat is maintained by frequent fire (Parr & Andersen 2008; Andersen et al. 2014). In contrast, *Oecophylla* is a foliage-nesting, forest-adapted taxon that favours shady habitats; populations can suffer significant mortality during fire, and frequent fire reduces its habitat suitability (Parr and Andersen 2008; Andersen et al. 2014). Fire can, therefore, be expected to regulate the relative abundance of these dominant taxa as partners in interactions with plant-based liquid carbohydrate.

Our study documents the ant–hemipteran and ant–EFN partners at our site, along with the relative incidence of ant–honeydew vs ant–EFN interactions, and then asks the following key questions. First, to what extent do behaviourally dominant dolichoderines and formicines (highest quality mutualists) visit honeydew over extrafloral nectar? Second, do the incidences of ant–honeydew and ant–EFN interactions vary among different fire regimes? Third, do honeydew or extrafloral nectar preferences of behaviourally dominant dolichoderines and formicines vary among fire regimes, thereby altering the relative incidence of higher quality mutualists? Finally, does frequent fire result in a switching of behaviourally dominant ant partners from forest-adapted *Oecophylla* to arid-adapted *Iridomyrmex*?

## Materials and methods

### Study site

The study was conducted in tropical savanna woodland at the Territory Wildlife Park, 40 km southeast of Darwin (12°42′ S 130°59′ E), Northern Territory, in the Australian seasonal tropics. The mean daily minimum temperature is 23.2 °C and maximum is 31.0 °C, while the mean annual rainfall is approximately 1700 mm (nearest station Darwin airport, Bureau of Meteorology), occurring primarily during a summer wet season (November-February). The vegetation of the study site is dominated by *Eucalyptus tetrodonta* F. Muell. and *Eucalyptus miniata* A.Cunn. ex Schauer over a ground-layer dominated by annual and perennial grasses (Scott et al. 2009). A diverse array of shrubs is also present, with EFN-bearing species of *Acacia* being especially common.

**Fig. 1:**
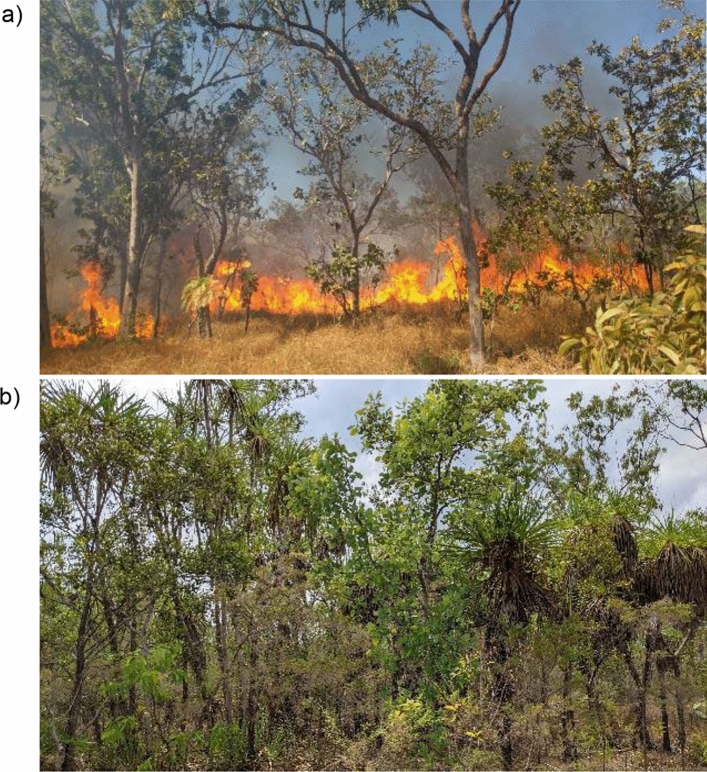
Examples of the different fire treatments at the Territory Wildlife Park near Darwin in Australia’s Northern Territory showing **a** an experimental fire during June 2020 at plot E2-B (burnt every 2 years since 2004), and **b** plot U-B (unburnt for > 30 yrs). The tallest trees are approximately 15 m in height. [Photo credits: A. Andersen]

Our study was conducted on 12 1-ha experimental plots that form part of a long-term burning experiment (Fig. 1; Fig S1). The experiment has six burning treatments, each with three replicate plots arranged in a randomised block design, that have been applied since 2004 (Nicholson et al. 2006; Parr et al. 2007; Andersen et al. 2014). For this study, we used four of these treatments, with three replicates each: E2—plots burned early during the dry season (May/June) every 2 years; E3—plots burned early during the dry every three years; L2—plots burned late during the dry season (September/October) every 2 years; and U—plots unburnt for > 25 years. The two treatments not included were E1 (burning early during the dry season each year) and E5 (early burning every 5 years), which were excluded due to logistical constraints. Woody cover varies markedly among the plots, decreasing with increasing fire frequency (Levick et al. 2019; Fig. 1a, b) and fire intensities are higher in the late compared with early fires. We refer to the replicated plots of each treatment as A, B and C, corresponding to the blocks in which they occur.

### Sampling

In each plot, we selected ten individuals (1.5–2 m in height) of each of four common plant species across the genera *Eucalyptus* (always *E. tetrodonta* F. Muell. and usually also *E. miniata* A.Cunn. ex Schauer; not possessing EFNs) and *Acacia* (usually *A. lamprocarpa* O. Schwarz and *A. dimidiata* Benth; all possessing EFNs) (Table S1). We selected individuals ≤ 2 m in height so that we could make observations on whole plants, and this meant that the eucalypts were all juveniles. It was not possible to find the same four plant species in all plots, although *Eucalyptus tetrodonta* occurred in all 12 plots, *Acacia lamprocarpa* in 11 plots and *E. miniata* in 10 plots (Table S1). In most plots, plants were selected from two species each of *Eucalyptus* and *Acacia*, but in two plots, plants were selected from one species of *Eucalyptus* and three species of *Acacia* (Table S1). There were thus 220 study plants of *Eucalyptus* and 260 of *Acacia* in total. Each plant within a plot was separated by at least 10 m such that most attendant ants on different plants were likely from different colonies (e.g. Agosti and Alonso, 2000; Oliveira et al. 2021).

**Fig. 2:**
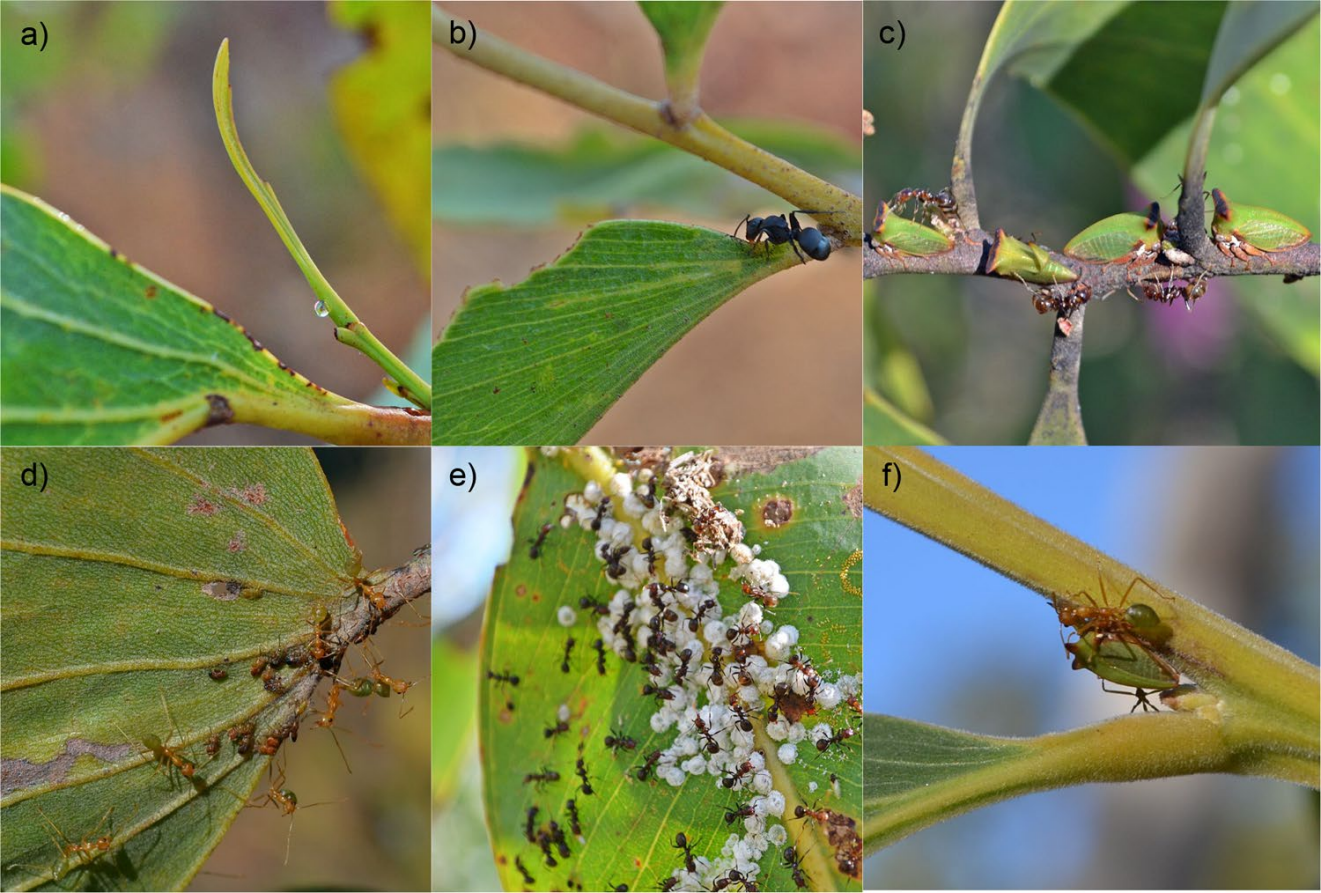
Images showing ant–honeydew and ant–extrafloral nectar mutualistic interactions: **a** an extrafloral nectar secretion on *Acacia holosericea,*, **b** *Polyrhachis inconspicua* interacting with extrafloral nectary on *Acacia oncinocarpa*, **c** *Papyrius* sp. 1 interacting with *Sextius virescens* (family Membracidae) on *Acacia* sp.*,* **d** *Oecophylla smaragdina* interacting with *Acizzia* nymphs (family Psyllidae) on *Acacia dimidiata*, **e** *Papyrius* sp. 1 interacting with *Glycaspis* lerps (family Aphalaridae) on *Eucalyptus miniata*, and **f** *Oecophylla smaragdina* interacting with *Sextius virescens* on *Acacia* sp. at the Territory Wildlife Park near Darwin in Australia’s Northern Territory. [Photo credits: F.M.P. Oliveira]

We observed each plant for two 10-min periods to record ants with different activity times throughout the day, one during the morning (7.00–10.00 am) and the other during the late afternoon/evening (4.00–7.00 pm) in July 2016. The two time periods for a plot were on separate days, with observations being made on a total of 12 days. We recorded all ants that were directly tending (mouthparts in contact with) honeydew-producing Hemiptera (for both *Eucalyptus* and *Acacia* species) or an EFN (*Acacia* species only) (Fig. 2) and collected ant and hemipteran specimens for later identification. Ants were identified to species following the nomenclature of previous ant studies at the study site (Parr et al. 2007; Andersen et al. 2014) and were classified into one of three dominance-based functional groups: dominant (species of *Iridomyrmex*, *Papyrius* and *Oecophylla*), subdominant (species of *Crematogaster* and *Monomorium*), or submissive (all other species), following Arnan et al. (2011). Hemipteran specimens were identified to family only. Vouchers of all taxa are held at the CSIRO laboratory in Darwin, Australia.

### Data analysis

Data from the two sampling periods of the day were pooled for each plot for all analyses, and ant species tending the same resource on both occasions were counted as two interactions. Cases of multiple ants from the same species tending the same resource were considered as single interactions (Câmara et al. 2018), and multiple species occurring at the same time at the same resource were counted as multiple interactions. We differentiated three resource types in our analyses: eucalypt honeydew, acacia honeydew and acacia EFN. To investigate the incidence of ant–honeydew vs ant–EFN interactions in the study system, we built a general linear mixed model (GLMM) using resource type as the explanatory variable. We considered as incidence of ant–honeydew and ant–EFN interactions (variable response) the number of interactions weighted by the number of observed plants per plot, since the number of both *Acacia* (with ant–honeydew and ant–EFN interactions) and *Eucalyptus* (with only ant–honeydew interactions) plants selected varied among plots (Table S1).

We built a GLMM to analyse how the incidence of ant–honeydew vs ant–EFN interactions vary among different fire regimes, using the relative incidence of these interactions as response variable, and fire regime and the interaction between fire regime and resource type as explanatory variables. We also built a GLMM to investigate the effects of long-term fire regimes on preferences of behaviourally dominant dolichoderines and formicines for honeydew over extrafloral nectar, using the relative incidence of ant interactions mediated by these ant functional groups per plot as the response variable, and the interaction between fire and functional group (dominant, subdominant, and submissive) as explanatory variables. To investigate if the relative incidence of behaviourally dominant (higher quality mutualists) and subordinate (lower quality mutualists) is affected by fire regime, we built a GLMM using the ratio of submissive: dominant relative incidence of interactions as the response variable.

To investigate if frequent fire results in a switching of behaviourally dominant ant partners from forest-adapted *Oecophylla* to arid-adapted *Iridomyrmex*, we built a GLMM using ratio of *Iridomyrmex*:*Oecophylla* incidence of interactions as the response variable, and fire regime as the explanatory variable. For all analyses involving fire, we repeated the GLMMs using two approaches: (1) comparisons among all individual burning treatments and (2) comparison between frequently burnt (E2 + L2 + E3) vs unburnt (U).

Given that *A. lamprocarpa* was sampled in all but one plot, we also conducted all analyses using just this species. For *A. lamprocarpa* models, we used the number of interactions rather than the incidence because each plot contained the same number (ten) of plants. In these models, we used a Poisson error distribution, block as random factor and checked for overdispersion. For the models using incidence of interactions as the response variable, we used a Gaussian error distribution and block as random factor, and we checked residuals for normality and homoscedasticity. Analyses were performed in the R software (R Core Team 2016).

## Results

### Overview

We recorded a total of 343 ant interactions, 128 with honeydew on *Eucalyptus*, 163 with honeydew on *Acacia* and 52 with EFNs on *Acacia*. Ten of the 46 *Acacia* plants with an ant–EFN interaction also had an ant–hemipteran interaction. The proportion of plants with ant–honeydew interactions was 36.3% for *Eucalyptus* and 44.6% *Acacia*, but the proportion of *Acacia* plants with ant–EFN interactions was much lower (18.9%). There was no significant difference in the incidence of ant–honeydew interactions on *Eucalyptus* (0.55 ± 0.42; mean ± SD) and *Acacia* (0.63 ± 0.34), but the incidence of ant–EFN interactions on *Acacia* was significantly lower than that of ant–honeydew interactions considering all *Acacia* species (0.27 ± 0.23; GLMM; *χ*^2^ = 12.80, df = 2, *p* < 0.001; Fig. S2). This trend was also observed when considering the number of interactions for *A. lamprocarpa* alone (Honeydew: 5.72 ± 3.58; EFN: 1.61 ± 2.38; GLMM; *χ*^2^ = 11.47, df = 2, *p* < 0.001; Fig. S3).

The number of interactions per plot did not vary significantly among plant species for either honeydew (*F* = 0.39, *p* = 0.89; species of both *Eucalyptus* and *Acacia*) or extrafloral nectar (*F* = 0.99, *p* = 0.46; species of *Acacia* only). This indicates that plot differences were not significantly influenced by differences in plant species. The incidence of multiple ant species at a single resource was low, occurring in only 8.2% of the ant–hemipteran interactions and 3.8% of ant–EFN interactions.

#### Ant–hemipteran and ant–EFN interactions

We recorded a total of 19 ant species from 10 genera collecting honeydew and/or extrafloral nectar (Table 1). Thirteen species from 9 genera collected honeydew, predominantly the behaviourally dominant *Oecophylla smaragdina* (56.3% of total interactions), *Iridomyrmex* sp. 21 (18.6%) and *Papyrius* sp. 1 (10.3%) (Table 1). Similarly, 13 ant species from 9 genera collected extrafloral nectar, with *O. smaragdina* (37.1% of total interactions) and *Iridomyrmex* sp. 21 (30.6%) also the most common (Table 1).

**Table 1:** Ant species tending honeydew and extrafloral nectaries across the 12 experimental burn plots at the Territory Wildlife Park near Darwin, Australia. Dominance hierarchy follows Arnan et al. (2011) and species are highlighted by dominance with underline bold = dominant, bold = subdominant, and no fill = submissive. HON = number of ant–honeydew interactions and EFN = number of ant–extrafloral nectar interactions

Subfamily/species	Dominance hierarchy	HON	EFN	Nº plots
*Dolichoderinae*				
***Iridomyrmex* sp. 21 (*minor* (Forel, 1915) complex)**	**Dominant**	**51**	**14**	**10**
***Iridomyrmex pallidus* (Forel, 1901)**	**Dominant**	**2**	**0**	**2**
***Iridomyrmex sanguineus* (Forel, 1910)**	**Dominant**	**4**	**2**	**1**
***Iridomyrmex* sp. 1 (*anceps* (Roger, 1863) complex)**	**Dominant**	**2**	**0**	**2**
*Ochetellus *sp. 2	Submissive	0	1	1
***Papyrius* sp. 1**	**Dominant**	**31**	**3**	**5**
*Ectatomminae*				
*Rhytidoponera aurata* (Roger, 1861)	Submissive	0	1	1
*Formicinae*				
*Camponotus* sp. 5 (*pellax* Santschi, 1919 gp.)	Submissive	3	0	2
***Oecophylla smaragdina* (Fabricius, 1775)**	**Dominant**	**167**	**21**	**11**
*Opisthopsis haddoni* (Emery, 1893)	Submissive	6	0	4
*Polyrhachis cyrus* (Forel, 1901)	Submissive	2	3	2
*Polyrhachis inconspicua* (Emery, 1887)	Submissive	0	1	1
*Polyrhachis schenckii* (Forel, 1886)	Submissive	0	1	1
*Polyrhachis senilis* (Forel, 1902)	Submissive	2	0	1
*Polyrhachis* sp. 12 (*obtusa* Emery, 1897 gp.)	Submissive	0	1	1
*Myrmicinae*				
***Crematogaster* sp. 2 (*australis* Mayr, 1876 complex)**	**Subdominant**	**0**	**1**	**1**
***Crematogaster* sp*.* 18 (*australis* Mayr, 1876 complex)**	**Subdominant**	**11**	**2**	**5**
***Monomorium* sp. 8 (*carinatum* Heterick, 2001 gp.)**	**Subdominant**	**8**	**0**	**2**
*Ponerinae*				
*Odontomachus* nr. *turneri* Forel, 1900	Submissive	1	1	1

The honeydew-producing Hemiptera represented six families from three superfamilies (Table S2). The most common family was Psyllidae, accounting for nearly half (44.5%) of the total number of hemipteran interactions and occurring on all eight plant species, followed by Cicadellidae (30.3%; seven) and Membracidae (21.7%; eight) (Table S2). Psyllidae and Cicadellidae were the most tended hemipterans by all ants regardless of functional group (Table 2). Cicadellidae occurred primarily on the eucalypts, whereas the other families were relatively evenly spread across plant genera (Table S2).

**Table 2:** Number of plants hosting honeydew-producing hemipteran and number of ant interactions (in brackets) considering each ant and Hemipteran taxa on the 12 experimental burn plots at Territory Wildlife Park, Darwin, NT. Ants are highlighted by dominance with underline bold  = dominant, bold = subdominant, and no fill = submissive. Ant codes represent Cam = *Camponotus* sp. 5, Cre = *Crematogaster* sp. 18, Iri21 = *Iridomyrmex* sp. 21, Irip = *Iridomyrmex pallidus*, Iris = *Iridomyrmex sanguineus*, Iri1 = *Iridomyrmex* sp. 1*,* Mon = *Monomorium* sp. 8, Odo = *Odontomachus nr. turneri*, Oec = *Oecophylla smaragdina*, Opi = *Opisthopsis haddoni,* Pap = *Papyrius* sp. 1*,* Polc = *Polyrhachis cyrus,* Pols = *Polyrhachis senilis*

Hemipteran families	Ant species												
	Cam	**Cre**	**Iri21**	**Irip**	**Iris**	**Iri1**	**Mon**	Odo	**Oec**	Opi	**Pap**	Polc	Pols
Aphalaridae	0	**0**	**0**	**0**	**0**	**0**	**0**	0	**3 (3)**	0	**0**	0	0
Psyllidae	1 (1)	**7 (7)**	**19 (23)**	**2 (2)**	**1 (1)**	**0**	**1 (2)**	0	**49 (68)**	4 (4)	**13 (19)**	1 (1)	1 (1)
Cicadellidae	2 (2)	**1 (1)**	**15 (18)**	**0**	**3 (3)**	**2 (2)**	**5 (6)**	1 (1)	**39 (44)**	2 (2)	**9 (10)**	0	0
Membracidae	0	**3 (3)**	**7 (8)**	**0**	**0**	**0**	**0**	0	**31 (50)**	0	**1 (1)**	0	0
Fulgoroidea	0	**0**	**3 (3)**	**0**	**0**	**0**	**0**	0	**2 (2)**	0	**1 (1)**	0	1 (1)

#### Ant functional composition and resource type

Behaviourally dominant species were numerically dominant at all resource types, but more so with honeydew (86.7% and 90.1% of total interactions for *Eucalyptus* and *Acacia*, respectively) than (*Acacia*) EFNs (76.9%; Table 1). Dominant ants were also the most common functional group across all honeydew-producing hemipteran families (Table 2). Submissive species were involved in just 3.5% of the interactions with honeydew, but in 17.3% of the interactions with EFNs (GLMM; *χ*^2^ = 6.19, df = 2, *p* = 0.045). For *A. lamprocarpa*, the ratio of submissive to dominant interactions was, on average, 97% higher with EFNs than with honeydew (GLMM; *χ*^2^ = 6.33; df = 1, *p* = 0.011).

### Effects of fire

**Fig. 3:**
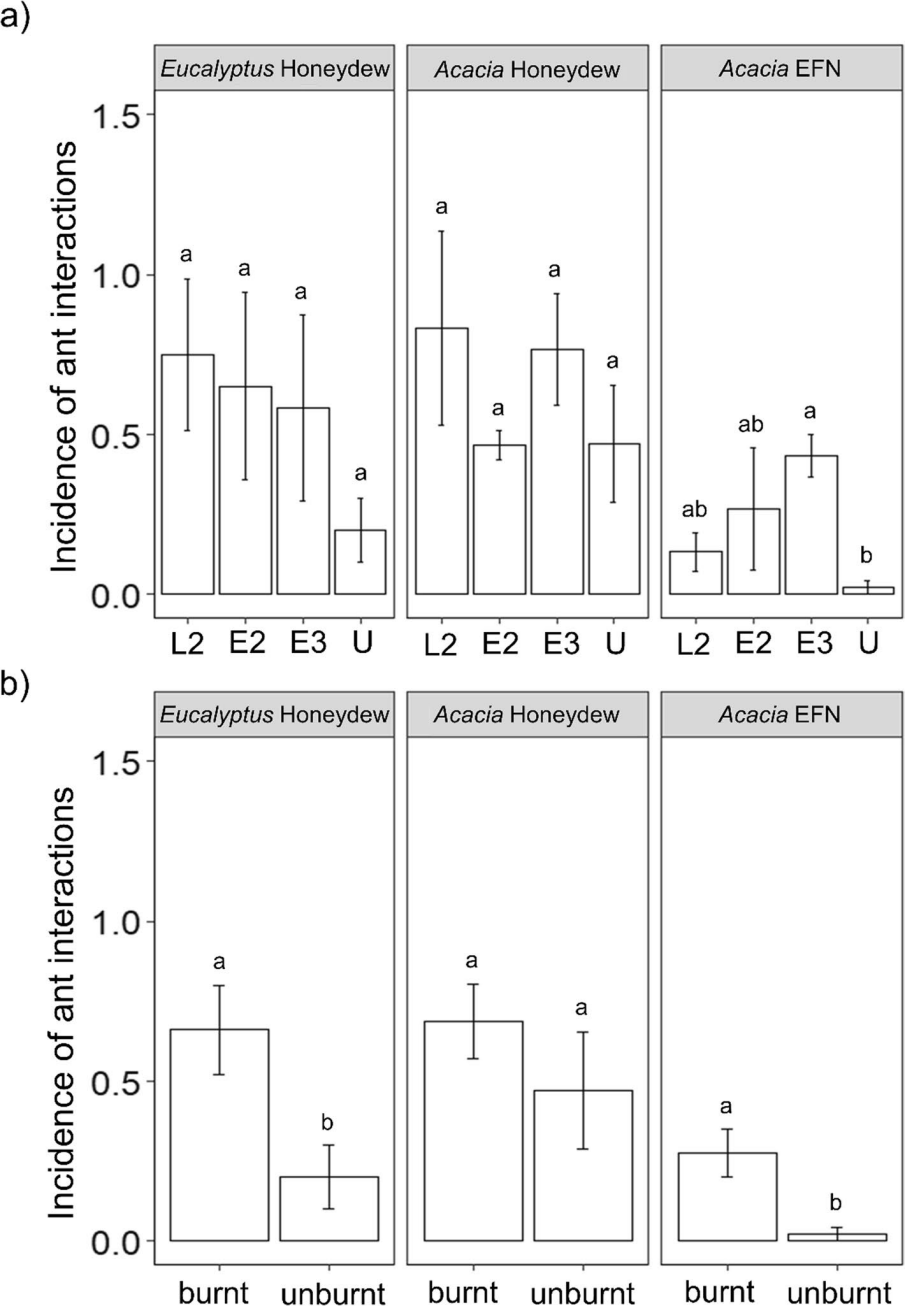
Mean incidence of ant interactions with eucalypt honeydew, acacia honeydew and acacia EFN across the 12 experimental burn plots at Territory Wildlife Park, Darwin, Australia. Comparisons of individual burning treatments (**a**) and frequently burnt (E2 + L2 + E3) vs unburnt plots (**b**). Codes represent plots with different fire frequency and time-since-fire: burning every 2 years late in the dry season (L2), burning every 2 years early in the dry season (E2), burning every 3 years early in the dry season (E3) and remaining unburnt (U). Different letters represent significant differences between fire regimes

When considering all fire treatments individually, the incidences of interactions involving both honeydew and EFNs were always lowest in unburnt plots, but the only statistically significant difference was between triennially burnt (E3) and unburnt in the incidence of (*Acacia*) EFN interactions (GLMM; *χ*^2^ = 20.11, df = 8, *p* = 0.009; Fig. 3a). For both resource types, incidence was significantly higher in burnt (E2 + L2 + E3) compared with unburned plots (Fig. 3b). In particular, ant–EFN interactions occurred on only 2.8% of *Acacia* plants on unburnt plots compared with 23.7% on burnt plots (GLMM; *χ*^2^ = 16.55, df = 4, *p* = 0.002; Fig. 3b). In contrast, the incidence of interactions with *Acacia* honeydew did not vary among individual fire treatments, nor between burnt and unburnt plots (Fig. 3). Similar patterns were found for *A. lamprocarpa*, with the number of ant–EFN interactions always lowest in unburned (U) plots, both considering fire treatment individually (GLMM; *χ*^2^ = 11.90; df = 8, *p* = 0.018; Fig. S4a) or burnt vs unburnt (GLMM; *χ*^2^ = 13.90; df = 4, *p* < 0.001; Fig. S4b).

**Fig. 4:**
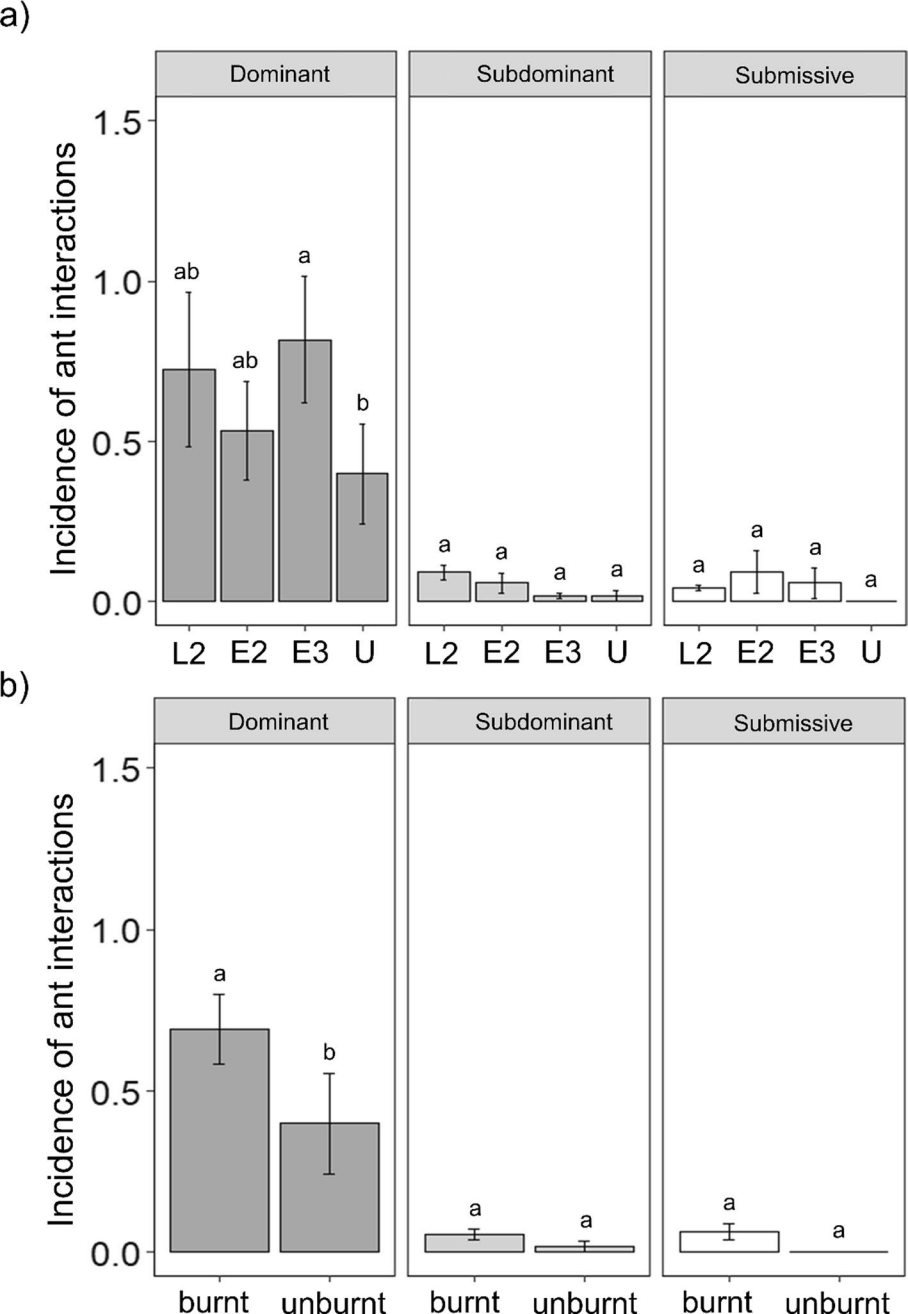
Mean incidence of ant interactions mediated by dominant, subdominant and submissive ants visiting honeydew and EFN resources (combined across *Eucalyptus* and *Acacia*) across the 12 experimental burn plots at Territory Wildlife Park, Darwin, Australia. Comparisons of within the individual burning treatments (**a**) and frequently burnt (E2 + L2 + E3) vs unburnt plots (**b**). Codes represent plots with different fire frequency and time-since-fire: burning every 2 years late in the dry season (L2), burning every 2 years early in the dry season (E2), burning every 3 years early in the dry season (E3) and remaining unburnt (U). Different letters represent significant differences between the fire regimes

We found a significant interactive effect of fire regime and functional group on the incidence of ant interactions considering both burning treatments individually (GLMM; *χ*^2^ = 7.02, df = 11, *p* < 0.001) and burnt compared with unburnt plots (GLMM; *χ*^2^ = 16.02, df = 5, *p* < 0.001). There was significant variation in the incidence of interactions involving dominant ant species but not either subdominant or subordinate ants (Fig. 4). More specifically, the incidence of interactions with dominant ants was higher in infrequently burnt (E3) than unburnt plots (Fig. 4a) and more generally in frequently burned than in unburnt plots (Fig. 4b). We did not detect significant effects of fire on the relative importance of submissive and dominant ants considering all burning treatments individually (GLMM; *χ*^2^ = 3.62, df = 3, *p* = 0.305; Fig. S5a). However, the relative importance of submissive ants was significantly higher in burnt than unburned plots (GLMM; *χ*^2^ = 4.27, df = 1, *p* = 0.038; Fig. S5b); this was because the proportional increase in burned vs unburned plots was higher for submissives compared with dominants, reflecting the almost absence of interactions involving submissives in unburnt plots. Considering the results for *A. lamprocarpa* alone, we found no effects of fire on the relative importance of submissive and dominant ants, either considering the fire treatments individually (GLMM; *χ*^2^ = 1.04, df = 3, *p* = 0.791) or when comparing burnt with unburnt plots (GLMM; *χ*^2^ = 0.036, df = 1, *p* = 0.899).

We detected no significant effects of fire on the relative importance of *Iridomyrmex* and *Oecophylla* as ant associates of either honeydew or EFNs, considering either all burning treatments individually (GLMM; *χ*^2^ = 3.62, df = 3, *p* = 0.305) or burnt compared with unburnt plots (GLMM; *χ*^2^ = 0.17, df = 1, *p* = 0.674). This was also the case for just *A. lamprocarpa* (GLMM; *χ*^2^ = 2.60, df = 3, *p* = 0.457; and GLMM; *χ*^2^ = 0.23, df = 1, *p* = 0.626, respectively).

## Discussion

Interactions between ants and liquid carbohydrates on plants are ubiquitous mutualisms whereby ants receive a key food resource in exchange for providing protection services. Liquid carbohydrate is a particularly important resource for behaviourally dominant ants because it energises their high levels of aggression, a trait that provides the highest quality protection services (Blüthgen et al. 2000; Davidson et al. 2004; Holway et al. 2002; Lach et al. 2020). Our study is the first comprehensive assessment of the incidence of ant interactions with honeydew versus extrafloral nectar in a highly diverse tropical system and is the first study of the effects of experimental fire regimes on such interactions.

### Incidence of honeydew, extrafloral nectar and dominant ants

We found that the incidence of ant–honeydew interactions on EFN-bearing species of *Acacia* was more than twice that of ant–EFN interactions, and a similarly high incidence of ant–honeydew interactions was observed in species of *Eucalyptus*. Our data, therefore, support previous findings that ants have a marked preference for honeydew over extrafloral nectar (Blüthgen et al. 2000; Buckley 1983; Campos and Camacho 2014; Fiala 1990; Sendoya et al. 2016). Our data are also consistent with honeydew being a primary food source for the numerically dominant ants because it is generally more abundant, nutritious, and constantly available (Blüthgen et al. 2000, 2004a, Fiala, 1990). Honeydew is particularly attractive to behaviourally dominant ants, which aggressively defend the resource and thereby monopolise it (Blüthgen et al. 2000). In our study, behaviourally dominant species of *Oecophylla*, *Iridomyrmex* and *Papyrius* comprehensively dominated honeydew resources, accounting for 90% of interactions with them despite representing just 5% of ant species recorded. These same species also dominated EFNs but to a lesser extent, indicating a preference for honeydew over extrafloral nectar. In contrast, the proportional incidence of submissive species at EFNs was five times higher than at honeydew-producing hemipterans. This can be attributed to the lower monopoly of EFNs by dominant species, which allows opportunistic access by other ants (Bluthgen et al. 2004b, Bluthgen and Fiedler 2004, Camacho and Campos, 2014, Davidson et al. 2003; Fagundes et al. 2016).

Notably, the primacy of honeydew as a source of liquid carbohydrate documented here does not hold in the Neotropics, either in savannas or epiphytes and lianas from Amazonian rainforest (Blüthgen et al. 2000; Camarota et al. 2015; Fagundes et al. 2016). For example, only 3% of trees at a site in Brazilian *cerrado* supported honeydew-producing hemipterans (compared with about 40% for the species of *Eucalyptus* and *Acacia* in our study), and this included just two of 861 trees bearing EFNs (Ribeiro et al. 2018). This striking biogeographical contrast in the relative importance of honeydew vs extrafloral nectar reflects a corresponding biogeographical contrast in the abundance of behaviourally dominant ants. Behaviourally dominant ants, especially dolichoderines, are extraordinarily abundant in Australia and this has been linked to an unusually high supply of liquid carbohydrate in the form of honeydew (Andersen 2016; Andersen et al. 2012). In our study, behaviourally dominant species accounted for about 90% of ant interactions with honeydew and about 80% of interactions with EFNs. Behaviourally dominant ants are far less abundant in Brazilian savannas (Andersen and Vasconcelos 2022), where they account for just a fraction of ant–liquid carbohydrate interactions, which most commonly involve species of *Camponotus*, *Cephalotes* and *Crematogaster* (Camarota et al. 2015; Fagundes et al. 2016; Ribeiro et al. 2018). One consequence of a lower incidence of dominant ants is that it allows access to liquid carbohydrate by a greater diversity of ants, potentially promoting ant species co-existence (Blüthgen et al. 2000; Fagundes et al. 2016).

### Effects of fire

Australian savanna ant communities are especially resilient in relation to fire because they are dominated by arid-adapted taxa that favour open habitats (Andersen et al. 2014; Andersen 2019). In our study system, fire-mediated differences among ant communities are evident only between strongly contrasting frequently burnt (producing relatively low canopy cover) and infrequently burnt (relatively high canopy cover) regimes (Andersen et al. 2014; Brassard et al. 2023). Our results are consistent with this since we typically noted little or no effect of individual fire regimes on ant interactions but often an effect of burnt versus unburnt treatments.

Fire markedly increased the incidence of ant–honeydew interactions on *Eucalyptus* and with ant–EFN interactions on *Acacia*. The effect of fire was particularly evident for ant–EFN interactions, which occurred on < 3% of *Acacia* plants on unburnt plots compared with nearly a quarter on burnt plots. In contrast, the incidence of interactions with *Acacia* honeydew was not affected by fire. The effects of fire on the incidence of ant–nectar interactions potentially occur through a range of mechanisms. One is a change in partner insect populations. We have no information on how fire affects hemipteran populations in our study system. However, ant abundance and diversity at the study site are promoted by frequent fire (Andersen et al. 2014), which is potentially a contributing factor. The effects of fire potentially also occur through changes in feeding behaviour. For example, nectar might be a more-attractive resource for ants in burnt compared with unburnt habitats (Blüthgen et al. 2004a, Bluthgen and Fiedler 2004, Fagundes et al. 2016). However, any such changes are unknown. Finally, the effects of fire potentially occur through changes in nectar quantity or quality (Alves-Silva and Del-Claro 2013, 2014; Delgado et al 2022). The strongest effect of fire was on the incidence of ant–EFN interactions on *Acacia*, suggesting that the quantity and/or quality of extrafloral nectar was higher in burnt plots. The incidence of ant–honeydew interactions on *Acacia* was not affected by fire, thus supporting this conclusion.

Frequent fire promoted the incidence of interactions involving behaviourally dominant ant species, particularly ant–honeydew interactions on *Eucalyptus*. This might simply reflect the promotion by frequent fire of the abundance of behaviourally dominant ant species (Andersen et al. 2012; Arnan et al. 2006; Vasconcelos et al. 2017). However, it might also involve a cascading positive effect on hemipteran populations due to the enhanced protection services they receive (Camacho et al. 2022). Consequently, ant–honeydew associations increase their frequency in burnt areas in relation to unburnt areas, as in the case of interactions in *Eucalyptus*.

Despite fire being previously shown to have contrasting effects on populations of the two key behaviourally dominant ant taxa in our study system—positive for *Iridomyrmex* and negative for *Oecophylla* (Andersen et al. 2014), we detected no fire-mediated switch in the incidence of these partners for either honeydew or extrafloral nectar. The incidence of interactions with *Iridomyrmex* was lower at unburnt compared with burnt plots as expected, but there was no concomitant increase in the incidence of *Oecophylla*. This seemingly contradictory result can be explained by our study using individual plants rather than whole plots as the units of study. It suggests that fire exclusion favours *Oecophylla* by increasing the density of woody plants (and thereby ant colony size and/or number) rather than increasing their abundance per plant.

## Conclusion

Mutualistic interactions with ants are ubiquitous globally (Bronstein 2015) and have important ecological consequences for food web dynamics and the fitness of plant and insect hosts (Del-Claro et al. 2016; Eubanks and Styrsky 2006; Styrsky and Eubanks 2007). These mutualisms are often subject to fire but ours is the first system-wide study of how they are impacted. Our study examines the incidence and identity of mutualistic partners rather than mutualistic outcomes for the partners. However, the promotion by fire of the number of interactions along with the relative incidence of highest quality ant mutualists, suggests that the protective services offered by ants in our tropical savanna study system are enhanced by frequent fire. This presumably has benefits to the plant and hemipteran partners. However, we note that our study system is one of the most fire-prone on Earth and its biota is highly adapted to frequent fire. Our findings of a positive effect of frequent burning on ant–liquid carbohydrate mutualisms, therefore, cannot be readily generalised to less fire-prone biomes. This is particularly the case for temperate ecosystems that historically experience fire at decadal to century scales but are now experiencing increased fire severity under anthropogenic climate change (Boer et al. 2020; Goss et al. 2020; Nolan et al. 2020). Further research is required to understand the responses of ant–liquid carbohydrate mutualisms to fire in a range of ecosystems and how these responses are likely to be impacted by increased fire risk.

## Supplementary information

Below is the link to the electronic supplementary material.

Supplementary file1 (DOCX 349 KB)

## Declarations

### Data availability

The data will be deposited in Dryad repository.
