# Supplementary material for: Fire and ant interactions mediated by honeydew and extrafloral nectar in an australian tropical savanna
Source: Oecologia. 2024 Oct 5;206(1-2):175–86. doi: 10.1007/s00442-024-05628-6 (PMC11489274; doi:10.1007/s00442-024-05628-6)

**Fire and ant interactions mediated by honeydew and extrafloral nectar in an Australian tropical savanna**

**Journal:** Oecologia

**Authors:** Fernanda M. P. Oliveira^1^, Carlos H. F. Silva^1^, Melinda L. Moir^2^, Inara R. Leal^3^, Alan N. Andersen^4^

**Affiliations:** ^1^Programa de Pós-Graduação em Biologia Vegetal, Universidade Federal de Pernambuco, Recife, Brazil; ^2^Department of Primary Industries and Regional Development, South Perth, WA, Australia; ^3^Departamento de Botânica, Universidade Federal de Pernambuco, Recife, Brazil; ^4^Research School for the Environment and Livelihoods, Charles Darwin University, Darwin, NT, Australia.

**Corresponding author:** Alan N. Andersen; alan.andersen@cdu.edu.au; +61 8 8946 6197; Research School for the Environment and Livelihoods, Charles Darwin University, Darwin, NT, Australia. E-mail address: [alan.andersen@cdu.edu.au](mailto:alan.andersen@cdu.edu.au)

**Supplementary Material**

**Table S1 –** Plant species (each ‘X’ represents ten individuals) sampled across the twelve experimental burn plots at the Territory Wildlife Park near Darwin, Australia. Codes represent plots with different fire frequency and time-since-fire: burning every two years late in the dry season (L2), burning every two (E2), three (E3) years early in the dry season, and remaining unburnt (U) since 2004. Replicates of treatments are represented by ‘A’, ‘B’ and ‘C’.

| **Plant Species** | **Plots** | | | | | | | | | | | |
| --- | --- | --- | --- | --- | --- | --- | --- | --- | --- | --- | --- | --- |
|  | L2 | | | E2 | | | E3 | | | U | | |
|  | A | B | C | A | B | C | A | B | C | A | B | C |
| *Acacia auriculiformis* A.Cunn. ex Benth. |  | X |  |  |  |  |  |  |  | X | X |  |
| *Acacia dimidiata* Benth. | X | X | X | X | X | X | X |  |  |  |  | X |
| *Acacia holosericea* G.Don |  |  |  |  |  |  |  | X | X |  |  |  |
| *Acacia lamprocarpa* O.Schwarz | X | X | X | X | X |  | X | X | X | X | X | X |
| *Acacia latescens* Benth. |  |  |  |  |  |  |  |  |  | X |  |  |
| *Acacia oncinocarpa* Benth. |  |  |  |  |  | X |  |  |  |  |  |  |
| *Eucalyptus miniata* A.Cunn. ex Schauer | X |  | X | X | X | X | X | X | X |  | X | X |
| *Eucalyptus tetrodonta* F.Muell. | X | X | X | X | X | X | X | X | X | X | X | X |

**Table S2 –** Number of plants hosting honeydew-producing hemiptera and number of ant interactions (in brackets) considering each plant and hemipteran taxa on the twelve experimental burn plots at Territory Wildlife Park, Darwin, Australia. Aaur = *Acacia auriculiformis*, Adim = *Acacia dimidiata*, Ahol = *Acacia holosericea*, Alam = *Acacia lamprocarpa*, Alat = *Acacia latescens*, Aonc = *Acacia oncinocarpa,* Emin = *Eucalyptus miniata*, Etet = *Eucalyptus tetrodonta*. Fulgoroidea was represented by two families.

| **Superfamily** / Family | Plant species | | | | | | | |
| --- | --- | --- | --- | --- | --- | --- | --- | --- |
|  | Aaur | Adim | Ahol | Alam | Alat | Aonc | Emin | Etet |
| **Psylloidea** |  |  |  |  |  |  |  |  |
| Aphalaridae | 0 | 1 (1) | 0 | 0 | 0 | 0 | 1 (1) | 1 (1) |
| Psyllidae | 6 (7) | 21 (39) | 3 (3) | 15 (28) | 4 (4) | 1 (1) | 11 (23) | 13 (24) |
| **Cicadelloidea** |  |  |  |  |  |  |  |  |
| Cicadellidae | 2 (2) | 8 (9) | 3 (3) | 10 (11) | 2 (2) | 0 | 17 (22) | 27 (39) |
| Membracidae | 5 (8) | 4 (6) | 6 (8) | 9 (24) | 2 (2) | 2 (3) | 4 (6) | 5 (6) |
| **Fulgoroidea** | 0 | 1 (1) | 0 | 0 | 0 | 0 | 4 (5) | 1 (1) |

**Figure S1.** Schematic of the 1-ha experimental plots in the long-term burning experiment at the Territory Wildlife Park near Darwin, Australia. Gray-filled plots denote those used in the study, while white plots were excluded.

**
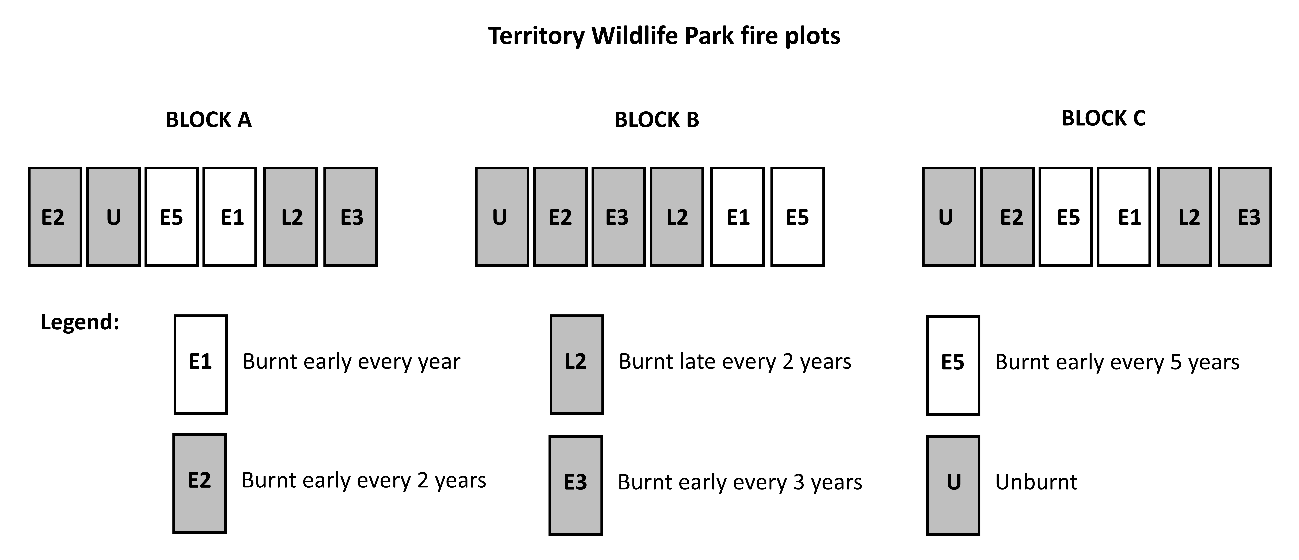
**

**Figure S2**. Mean incidence (total interactions divided by number of study plants) of ant-honeydew interactions on *Eucalyptus* and *Acacia* and ant-extrafloral nectar (EFN) interactions on *Acacia* across the twelve experimental burn plots at the Territory Wildlife Park near Darwin, Australia. Different letters represent significant differences between resource types.

**
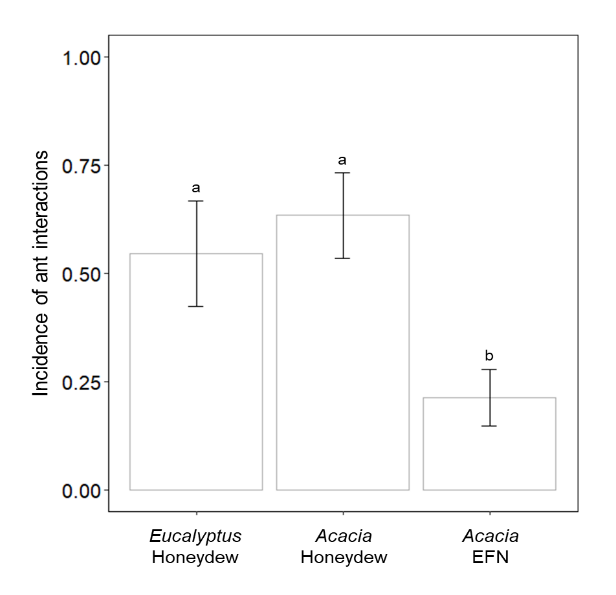
**

**
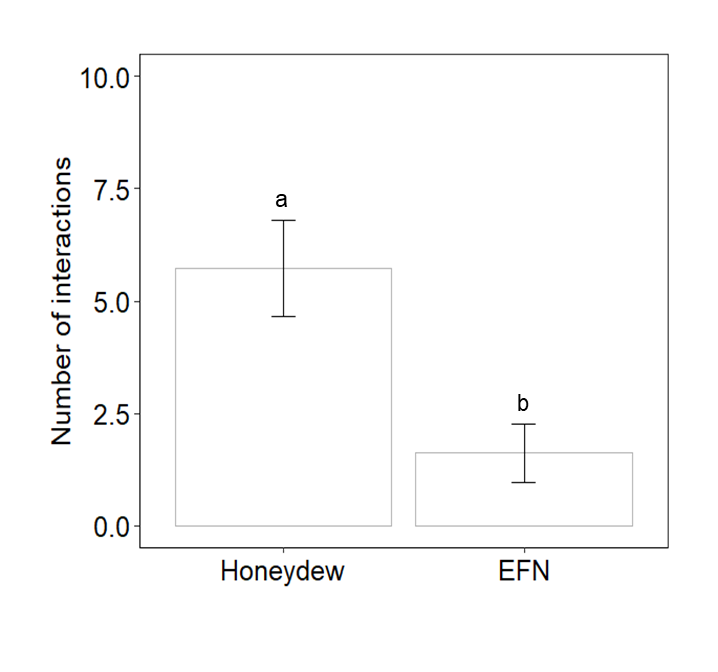
Figure S3**. Mean number of interactions of ant-honeydew interactions and ant-extrafloral nectar (EFN) interactions on *Acacia lamprocarpa* across the twelve experimental burn plots at the Territory Wildlife Park near Darwin, Australia. Different letters represent significant differences between resource types.

**Figure S4.** Mean number of interactions with *Acacia lamprocarpa* honeydew and extrafloral nectar (EFN) across the twelve experimental burn plots at Territory Wildlife Park, Darwin, Australia. Comparisons of individual burning treatments (a) and burnt (E2 + L2 + E3) vs unburnt plots (b). Codes represent plots with different fire frequency and time-since-fire: burning every two years late in the dry season (L2), burning every two (E2) and three (E3) years early in the dry season, and remaining unburnt (U). Different letters represent significant differences between fire regimes.

**
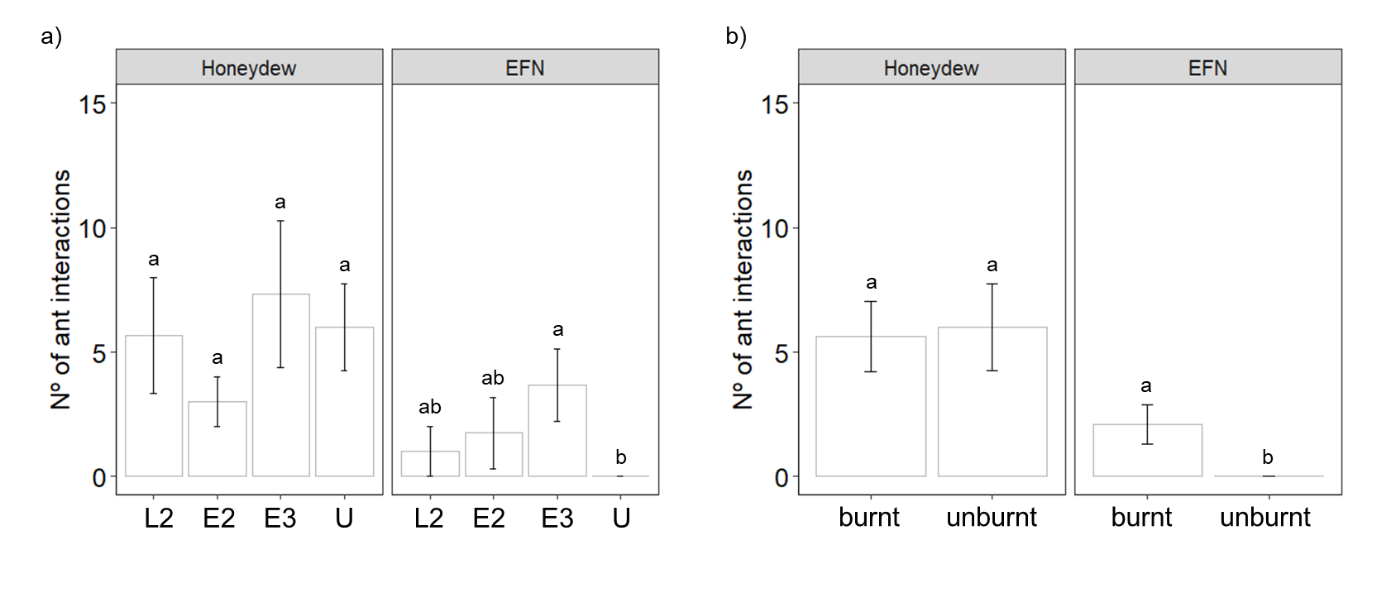
**

**Figure S5.** Mean ratio of interactions with submissive versus dominant ants visiting different resource types (honeydew on *Eucalyptus*, honeydew on *Acacia* and extrafloral nectar on *Acacia*) across the twelve experimental burn plots at the Territory Wildlife Park near Darwin, Australia. Comparations within the individual burning treatments (a) and frequently burnt (E2 + L2 + E3) vs unburnt plots (b). Codes represent plots with different fire frequency and time-since-fire: burning every two years late in the dry season (L2), burning every two (E2) and three (E3) years early in the dry season, and remaining unburnt (U). Different letters represent significant differences between functional groups and resource types.


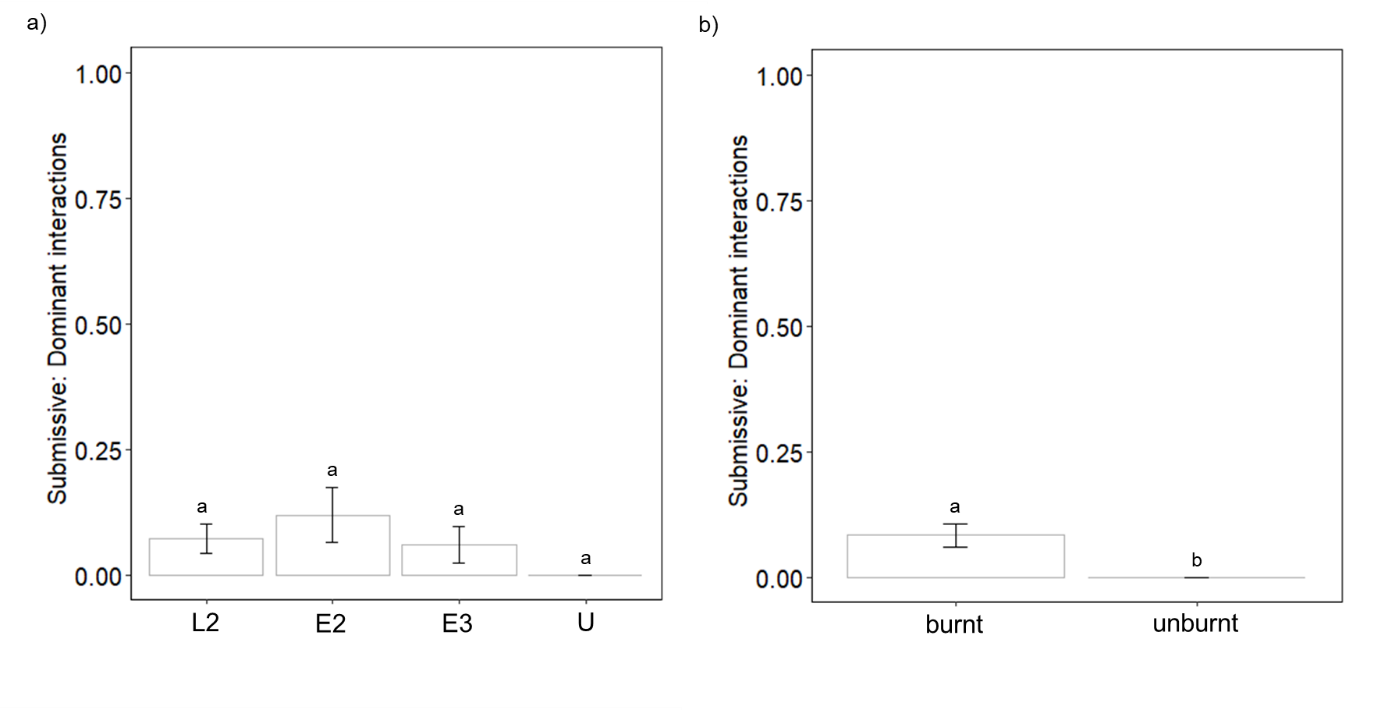

Supplement: Supplementary file 1 — Supplementary file1 (DOCX 349 KB) [file 442_2024_5628_MOESM1_ESM.docx]
